# Reconfiguring robotic surgery in head and neck practice: transition from multi-port to single-port with early clinical outcomes

**DOI:** 10.3389/fonc.2026.1858782

**Published:** 2026-07-10

**Authors:** Shih-Wei Chen, Ying-Lyung Hsiao, Chang-Yo Pan, Chuck Lin, Frank Cheau-Feng Lin, Mei-Wen Nian, Stella Chin-Shaw Tsai

**Affiliations:** 1 Department of Otolaryngology, Tungs’ Taichung MetroHarbor Hospital, Taichung, Taiwan; 2 Department of Stomatology, Tungs’ Taichung MetroHarbor Hospital, Taichung, Taiwan; 3 School of Medicine, Johns Hopkins University, Baltimore, MD, United States; 4 Department of Surgery, Chung Shan Medical University, Taichung, Taiwan; 5 Department of Thoracic Surgery, Chung Shan Medical University Hospital, Taichung, Taiwan; 6 Department of Medical Research, Tungs’ Taichung MetroHarbor Hospital, Taichung, Taiwan; 7 Department of Post-Baccalaureate Medicine, National Chung Hsing University, Taichung, Taiwan; 8 College of Life Sciences, National Chung Hsing University, Taichung, Taiwan

**Keywords:** early implementation, head and neck surgery, retroauricular approach, robotic neck surgery, single-port robotic surgery, transcervical approach, transoral robotic surgery

## Abstract

**Background:**

The integration of the da Vinci single-port (SP) platform into head and neck practice represents a major transition from conventional multi-port robotic systems, yet real-world data on early institutional adoption remain limited. This study evaluated the clinical integration of the SP platform during an early implementation period, with particular focus on case accrual, diversification of surgical access routes, surgeon-specific adoption patterns, and temporal evolution of case selection.

**Methods:**

This retrospective observational study included consecutive robotic head and neck procedures performed with the da Vinci SP system at a single institution during a 9-month transition period prior to the installation of a second SP system. Clinical, operative, and perioperative variables were extracted from an amended institutional dataset and operative records. Cases were analyzed overall, compared between the 2 primary surgeons, and chronologically stratified into Early and Later phases.

**Results:**

A total of 64 cases were performed, including 34 malignant (53.1%) and 30 benign (46.9%) lesions. Case accrual increased steadily throughout the study period. Transoral surgery was the most common access route (42/64, 65.6%), followed by transoral plus transcervical (12/64, 18.8%), transcervical alone (5/64, 7.8%), and retroauricular access (5/64, 7.8%). Surgeon 1 performed 51 cases and treated an older population with significantly higher rates of tobacco, alcohol, and betel quid exposure, more malignant disease, and broader anatomic distribution. Surgeon 2 performed 13 cases, all through a transoral approach, predominantly for benign lesions. A chronological comparison between the Early and Later phases showed stable surgeon distribution, pathology patterns, and approach patterns, but oral cavity cases and reconstructive procedures became more prominent in the Later phase. Cumulative accrual curves for benign and malignant disease progressed in parallel, suggesting that SP adoption expanded across both oncologic and benign indications rather than being confined to a single clinical niche.

**Conclusion:**

Early institutional integration of the da Vinci SP platform was associated with steady case accrual, expansion of operative access pathways, and broad adoption across malignant and benign head and neck indications. These findings provide real-world evidence that transition from multi-port to single-port robotic surgery can be achieved successfully within a high-volume head and neck program.

## 1 Introduction

The integration of single-port (SP) robotic systems into established multi-port platforms represents a critical step in the evolution of robotic head and neck surgery. The da Vinci SP system was specifically developed to address the ergonomic and technical limitations of single-incision surgery, offering improved cosmesis, reduced incision morbidity, and enhanced maneuverability within narrow anatomical spaces (1, 2).

Our institution previously validated the feasibility and safety of the da Vinci SP platform through a multi-specialty prospective observational study that included initial cases across urology, general surgery, gynecology, and otolaryngology (3). The early experience confirmed that the SP system could be deployed efficiently, achieving consistent docking and draping times of 5–6 minutes. While the otolaryngology cohort in that study (n=12) demonstrated excellent feasibility in confined anatomical regions, these patients initially required longer hospital stays (mean 13.8 days). We further established the platform’s utility and safety through a prospective real-world case series evaluating single-port transoral robotic surgery specifically for head and neck lesions and obstructive sleep apnea (OSA) (4).

Transitioning from this initial validation phase to routine clinical implementation necessitates a structured institutional learning curve, as operative metrics and efficiency typically require an accumulated volume of cases to stabilize. However, real-world implementation and its impact on surgical access strategies remain insufficiently characterized. Therefore, this study aimed to evaluate the clinical integration and reconfiguration of surgical approaches during a defined institutional transition from multi-port to single-port systems within a high-volume head and neck program.

## 2 Materials and methods

### 2.1 Study design and cohort

This retrospective observational study included a consecutive cohort of 64 patients who underwent robotic head and neck surgery using the da Vinci SP system at our institution during the early phase of platform implementation. All surgical procedures were performed by two board-certified head and neck surgeons (Surgeon 1 and Surgeon 2). At the initiation of the study, Surgeon 1 had 24 years of post-fellowship experience and had performed >500 multi-port robotic cases. Surgeon 2 had 15 years of post-fellowship experience and had performed >100 multi-port robotic cases.

The institutional transition period extended from May 23, 2025, to February 26, 2026, before installation of a second SP system. During this early implementation interval, standard da Vinci multi-port (Xi) robotic systems remained fully operational and available at our institution. The introduction of the SP platform did not represent an immediate or complete replacement of existing technology; rather, both multi-port and single-port platforms were utilized in parallel. The selection of the surgical platform was non-random and left entirely to the attending surgeon’s clinical discretion, based on patient anatomy, tumor characteristics, and the perceived technical advantages of the single-port configuration in specific anatomical corridors. The clinical cohort comprised procedures performed from May 24, 2025, to February 26, 2026. Surgical indications included head and neck malignancies, benign lesions, and obstructive sleep apnea. This study was approved by the Institutional Review Board of Tungs’ Taichung MetroHarbor Hospital (IRB# 1140099), which waived the requirement for written informed consent due to the retrospective nature of the data analysis.

### 2.2 Data collection and variable definition

Clinical data were retrospectively collected from the amended institutional dataset and operative records. Baseline variables included age, sex, tobacco use, alcohol exposure, betel quid use, pathological behavior, and anatomic site. Pathological behavior was classified as malignant or benign. Anatomic sites were grouped as oropharynx/tonsil, hypopharynx, larynx, oral cavity, salivary or parotid region, and neck or deep neck region.

Surgical approach was categorized into four groups: transoral, transoral combined with transcervical, transcervical, and retroauricular. Operative characteristics and perioperative adjuncts were abstracted from free-text operative notes and procedure records. These variables included concomitant procedures, such as reconstruction, dental extraction, and combined plastic surgery with free flap reconstruction. All reconstructive procedures (such as local muscle flaps or microvascular free flap insets) were performed manually using conventional open or transoral techniques after the robotic resection was completed. Reconstruction was included strictly as a variable to reflect baseline case complexity. Similarly, perioperative support measures, including nasogastric tube placement, intensive care unit (ICU) stay, and tracheotomy, were noted. Use of intraoperative adjuncts and materials, including tissue glue and facial nerve monitoring, was also recorded. Safety outcomes, as detailed rates for overall complications, major complications, intraoperative conversions, 30-day reoperations (take-back procedures), postoperative bleeding, wound infections, unplanned readmissions, and 30-day mortality, were assessed. Variables derived from free text fields were harmonized before analysis.

### 2.3 Chronological stratification

The temporal analysis was structured around a case-sequence-based learning curve assessment to compare the initial 32 cases to the subsequent 32 cases. The cohort of 64 cases was stratified into the Early vs. Later phase of this initial adoption period. According to the current dataset, the Early phase spanned May 23, 2025, to October 15, 2025, and the Later phase spanned October 16, 2025, to February 26, 2026. These phases were used to assess changes in case selection, surgical access patterns, and perioperative characteristics over time. Due to sample constraints, a robust cumulative sum (CUSUM) analysis was not performed.

### 2.4 Statistical analysis

Comparisons were performed between the two primary surgeons and between the Early and Later phases. Continuous variables were summarized as mean ± standard deviation or median with interquartile range, as appropriate. For between-group comparisons, the Mann–Whitney U test was used for continuous variables, Fisher’s exact test for binary categorical variables, and chi-square tests for categorical variables with more than two categories, as appropriate. A two-sided p-value of less than 0.05 was considered statistically significant. All statistical analyses were performed using SPSS Statistics version 28.0 (IBM Corp., Armonk, NY, USA) and R version 4.3.1 (R Foundation for Statistical Computing, Vienna, Austria).

## 3 Results

### 3.1 Case accrual during early SP implementation

**Figure 1:**
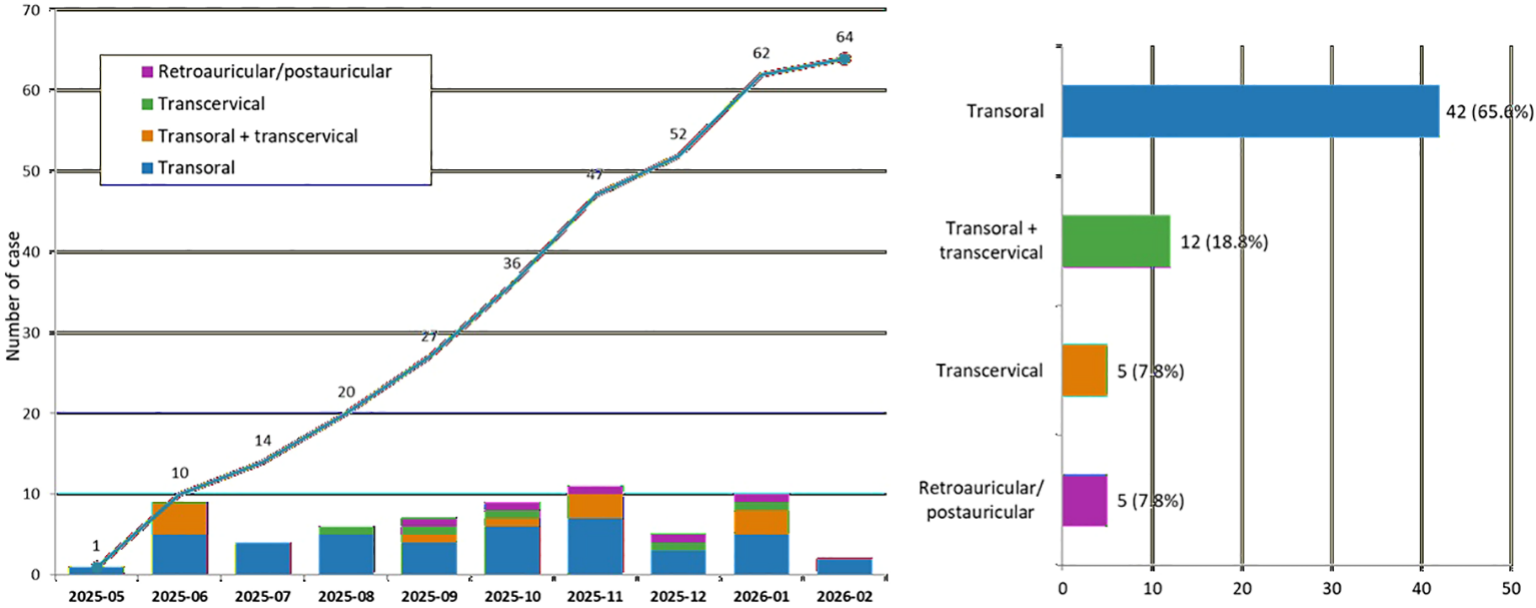
Chronological case accrual and monthly distribution of surgical approaches. Monthly case volume during early implementation of the da Vinci single-port (SP) system. Stacked bars indicate the surgical approach, and the line indicates cumulative case accrual.

As shown in **Figure 1**, a total of 64 robotic head and neck procedures were performed during the 9-month early implementation period of the da Vinci SP platform at our institution. The case accrual pattern reflects steady institutional adoption of the SP system throughout the study period, prior to the installation of a second SP platform. During this interval, Surgeon 1 performed 51 cases (79.7%), while Surgeon 2 performed 13 cases (20.3%).

### 3.2 Schematic distribution of operative access pathways

**Figure 2:**
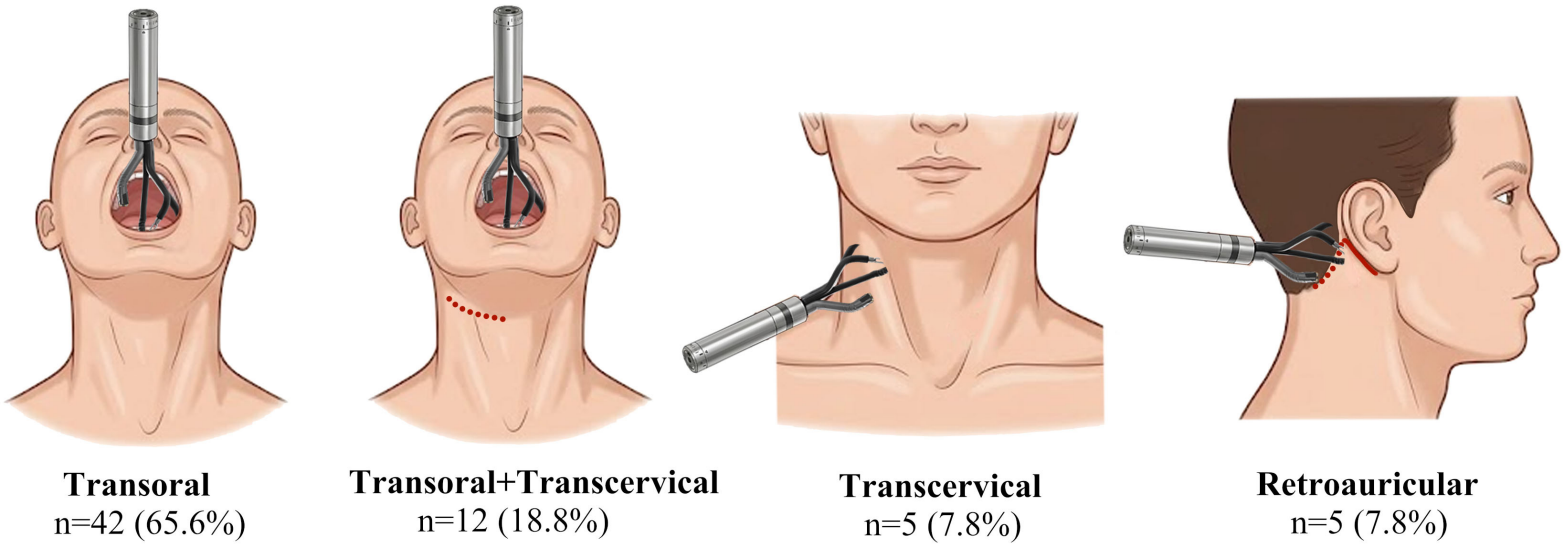
Schematic representation of surgical access routes in the 64-case da Vinci SP cohort. Illustration of the 4 operative access routes used in this cohort: transoral, transoral plus transcervical, transcervical, and retroauricular.

**Figure 2** provides a schematic representation of the surgical access routes used during this early implementation period. These included transoral, combined transoral and transcervical, transcervical, and retroauricular approaches. In terms of surgical access, transoral surgery was the dominant approach, accounting for 42 cases (65.6%), followed by transoral combined with transcervical access in 12 cases (18.8%), transcervical access alone in 5 cases (7.8%), and retroauricular access in 5 cases (7.8%). The figure illustrates that application of the SP platform extended beyond a single transoral corridor and was adapted to multiple anatomic entry routes in selected head and neck procedures.

### 3.3 Baseline characteristics of the implementation cohort

Baseline characteristics of the 64-case implementation cohort are summarized in **Table 1**. Overall, 34 cases (53.1%) were malignant and 30 (46.9%) were benign. The most common anatomic site was the oropharynx or tonsil (28/64, 43.8%), followed by the oral cavity (24/64, 37.5%), salivary or parotid region (6/64, 9.4%), hypopharynx (2/64, 3.1%), larynx (2/64, 3.1%), and neck or deep neck region (2/64, 3.1%).

**Table 1:** Baseline characteristics of the 64-case da Vinci SP implementation cohort.

Characteristic	Overall (N = 64)	Surgeon 1 (n=51)	Surgeon 2 (n=13)	*P* value
Age, years, median (IQR)	50.0 (42.0-59.0)	51.0 (46.0-62.0)	39.0 (31.5-51.5)	.040*
Sex				
Male	52 (81.2%)	42 (82.4%)	10 (76.9%)	.697
Female	12 (18.8%)	9 (17.6%)	3 (23.1%)	
Tobacco				
Yes	34 (53.1%)	32 (62.7%)	2 (15.4%)	.003*
No	30 (46.9%)	19 (37.3%)	11 (84.6%)	
Alcohol				
Yes	23 (35.9%)	23 (45.1%)	0 (0.0%)	.001*
No	41 (64.1%)	28 (54.9%)	13 (100.0%)	
Betel quid				
Yes	22 (34.4%)	22 (43.1%)	0 (0.0%)	.002*
No	42 (65.6%)	29 (56.9%)	13 (100.0%)	
Pathology behavior				
Malignant	34 (53.1%)	33 (64.7%)	1 (7.7%)	<.001*
Benign	30 (46.9%)	18 (35.3%)	12 (92.3%)	
Anatomic site				
Oropharynx	28 (43.8%)	16 (31.4%)	12 (92.3%)	.002*
Hypopharynx	2 (3.1%)	2 (3.9%)	0 (0.0%)	
Larynx	2 (3.1%)	1 (2.0%)	1 (7.7%)	
Oral cavity	24 (37.5%)	24 (47.1%)	0 (0.0%)	
Parotid	6 (9.4%)	6 (11.8%)	0 (0.0%)	
Neck/deep neck	2 (3.1%)	2 (3.9%)	0 (0.0%)	

SP, single-port; IQR, interquartile range. *P<0.05 denotes statistical significance.

When stratified by surgeon, Surgeon 1 performed 51 of 64 cases (79.7%), whereas Surgeon 2 performed 13 cases (20.3%). Patients treated by Surgeon 1 were significantly older than those treated by Surgeon 2, with a median age of 51.0 [IQR, 46.0 to 62.0] years versus 39.0 [IQR, 31.5 to 51.5] years, p = .040. Sex distribution did not differ significantly between the two surgeons.

Exposure-related risk factors differed significantly between the two surgeon cohorts. Tobacco use was more common in Surgeon 1’s patients than in Surgeon 2’s patients (62.7% vs. 15.4%, p = .003). Alcohol exposure (45.1% vs. 0.0%, p = .001) and betel quid use (43.1% vs. 0.0%, p = .002) were likewise more frequent in Surgeon 1’s cohort. In addition, malignant disease predominated in Surgeon 1’s practice (64.7%), whereas Surgeon 2 treated predominantly benign lesions (92.3%) (p <.001).

Anatomic site distribution also varied significantly between surgeons (p = .002). Surgeon 2’s cases were concentrated mainly in the oropharynx or tonsil, whereas Surgeon 1 managed a broader range of sites, including the oral cavity, salivary or parotid region, hypopharynx, larynx, and deep neck lesions.

### 3.4 Operative characteristics and perioperative adjuncts by surgeon

Operative characteristics and perioperative adjuncts are summarized in **Table 2**. All cases performed by Surgeon 2 used a transoral approach, whereas Surgeon 1 employed a broader range of access routes, including transoral combined with transcervical, transcervical alone, and retroauricular approaches. Accordingly, the distribution of surgical approach categories differed significantly between the two surgeons (p = .041).

**Table 2:** Operative characteristics and perioperative adjuncts in the 64-case da Vinci SP early implementation cohort.

Characteristic	Overall (N = 64)	Surgeon 1 (n=51)	Surgeon 2 (n=13)	P value
Approach category				
Transoral	42 (65.6%)	29 (56.9%)	13 (100.0%)	.041*
Transoral + Transcervical	12 (18.8%)	12 (23.5%)	0 (0.0%)	
Transcervical	5 (7.8%)	5 (9.8%)	0 (0.0%)	
Retroauricular	5 (7.8%)	5 (9.8%)	0 (0.0%)	
Concomitant procedure				
Reconstruction (rotation flap or STSG)	18 (28.1%)	18 (35.3%)	0 (0.0%)	.008*
Dental extraction	5 (7.8%)	5 (9.8%)	0 (0.0%)	.308
Combined plastic surgery (free flap)	8 (12.5%)	8 (15.7%)	0 (0.0%)	.144
Perioperative adjuncts/support				
Tracheotomy	0 (0.0%)	0 (0.0%)	0 (0.0%)	–
Nasogastric tube	23 (35.9%)	22 (43.1%)	1 (7.7%)	.015*
ICU stay	11 (17.2%)	11 (21.6%)	0 (0.0%)	.064
Devices/materials used intraoperatively				
Tissue glue	14 (21.9%)	14 (27.5%)	0 (0.0%)	.027*
Facial nerve monitor	6 (9.4%)	6 (11.8%)	0 (0.0%)	.333
Safety outcomes				
Any complication	13 (20.3%)	13 (25.5%)	0 (0.0%)	.054
Major complication	3 (4.7%)	3 (5.9%)	0 (0.0%)	1.00
Conversion to open	0 (0.0%)	0 (0.0%)	0 (0.0%)	–
Return to OR/reoperation	2 (3.1%)	2 (3.9%)	0 (0.0%)	1.00
Postoperative bleeding	3 (4.7%)	3 (5.9%)	0 (0.0%)	1.00
Wound infection	6 (9.4%)	6 (11.8%)	0 (0.0%)	.333
Re-admission	1 (1.6%)	1 (2.0%)	0 (0.0%)	1.00
Mortality	0 (0.0%)	0 (0.0%)	0 (0.0%)	–

ICU, intensive care unit; SP, single-port; STSG, split-thickness skin graft. *P<0.05 denotes statistical significance.

Concomitant procedures were more frequently performed in Surgeon 1’s cohort. Reconstruction was undertaken in 18 of 51 cases (35.3%) by Surgeon 1 but in none of the 13 cases performed by Surgeon 2 (p = .008). Combined plastic surgery with free flap reconstruction was observed only in Surgeon 1’s cases (15.7% vs. 0.0%), although this difference did not reach statistical significance. Dental extraction was likewise limited to Surgeon 1’s cohort. All concomitant procedures were performed manually following the completion of the robotic resection.

With respect to perioperative support, nasogastric tube placement was significantly more common in Surgeon 1’s cases than in Surgeon 2’s cases (43.1% vs. 7.7%, p = .015). Intensive care unit stay occurred in 21.6% of Surgeon 1’s cases and in none of Surgeon 2’s cases, showing a nonsignificant trend (p = .064). No patient underwent tracheotomy. Among intraoperative adjuncts, tissue glue was used more frequently by Surgeon 1 (27.5% vs. 0.0%, p = .027), whereas facial nerve monitoring was used only in Surgeon 1’s cases without a statistically significant difference between surgeons.

Safety and perioperative complication outcomes are detailed in **Table 2**. Overall, complications occurred in 13 patients (20.3%), all of whom were treated by Surgeon 1, representing a complication rate of 25.5% for Surgeon 1 and 0.0% for Surgeon 2 (p = .054). Major complications occurred in 3 patients overall (4.7%), all within Surgeon 1’s cohort (5.9% vs. 0.0%, p = 1.00). Postoperative bleeding and wound infections were observed in 3 (4.7%) and 6 (9.4%) patients, respectively, exclusively under the care of Surgeon 1. The 30-day reoperation rate was 3.1% (n = 2), with both cases requiring a return to the operating room for management of postoperative bleeding. There was 1 unplanned hospital readmission (1.6%) in the cohort, and no instances of intraoperative conversion to open surgery or 30-day mortality were recorded (0.0%).

### 3.5 Chronological evolution of case selection and access patterns

Chronological comparisons between the first 32 cases and the subsequent 32 cases are presented in **Table 3**. Patient age, surgeon distribution, and pathology behavior were similar between the two phases. Malignant disease accounted for 16 of 32 cases (50.0%) in the early phase and 18 of 32 cases (56.2%) in the later phase (p = .616). Likewise, the proportion of cases performed by Surgeon 1 remained stable over time (81.2% in the early phase vs. 78.1% in the later phase, p = .756).

**Table 3:** Chronological comparison of case characteristics between the early and later phases of da Vinci SP implementation.

Characteristic	Early phase (n=32)	Later phase (n=32)	P value
Study period	2025-05–24 to 2025-10-15	2025-10–16 to 2026-02-26	
Age, years, median (IQR)	47.5 (41.3-61.3)	51.0 (42.3-57.8)	.453
Surgeon			
1	26 (81.2%)	25 (78.1%)	.756
2	6 (18.8%)	7 (21.9%)	
Pathology behavior			
Malignant	16 (50.0%)	18 (56.2%)	.616
Benign	16 (50.0%)	14 (43.8%)	
Anatomic site			
Oropharynx/tonsil	17 (53.1%)	11 (34.4%)	.263
Hypopharynx	1 (3.1%)	1 (3.1%)	
Larynx	1 (3.1%)	1 (3.1%)	
Oral cavity	8 (25.0%)	16 (50.0%)	
Salivary/parotid	3 (9.4%)	3 (9.4%)	
Neck/deep neck	2 (6.3%)	0 (0.0%)	
Approach category			
Transoral	22 (68.8%)	20 (62.5%)	.843
Transoral + Transcervical	5 (15.6%)	7 (21.9%)	
Transcervical	3 (9.4%)	2 (6.2%)	
Retroauricular	2 (6.2%)	3 (9.4%)	
Operative profile markers			
Reconstruction	7 (21.9%)	11 (34.4%)	.202
Dental extraction	5 (15.6%)	0 (0.0%)	.026*
Combined plastic surgery	2 (6.3%)	6 (18.8%)	.128
Tracheotomy	0 (0.0%)	0 (0.0%)	–
Nasogastric tube	11 (34.4%)	12 (37.5%)	.500
ICU stay	6 (18.8%)	5 (15.6%)	.500

*P<0.05 denotes statistical significance.

The distribution of anatomic sites shifted over time, although the overall comparison was not statistically significant (p = .263). Oropharyngeal or tonsillar cases were more common in the early phase (53.1% vs. 34.4%), whereas oral cavity cases became more prominent in the later phase (50.0% vs. 25.0%). Surgical approach patterns remained broadly similar between phases (p = .843), with transoral surgery continuing to predominate in both the early and later phases (68.8% and 62.5%, respectively). Combined transoral and transcervical access increased modestly from 15.6% to 21.9%, whereas transcervical and retroauricular approaches remained relatively infrequent.

Among operative profile markers, reconstruction was more frequent in the later phase than in the early phase (34.4% vs. 21.9%), and combined plastic surgery also increased numerically (18.8% vs. 6.3%), although neither difference was statistically significant. In contrast, dental extraction was observed only in the early phase (15.6% vs. 0.0%, p = .026). Rates of nasogastric tube placement and intensive care unit stay were similar between phases, and no tracheotomy was performed in either phase.

**Figure 3:**
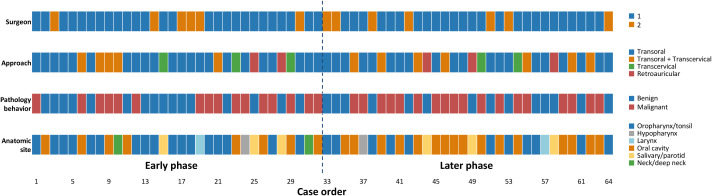
Case-order procedural map of the 64-case da Vinci SP cohort. Sequential distribution of cases by primary surgeon, surgical approach, pathology behavior, and anatomic site. The dashed line separates the early and later phases.

The temporal evolution is visualized in **Figure 3**, which maps each case chronologically by surgeon, access route, pathology behavior, and anatomic site. The figure shows that transoral surgery remained the dominant approach throughout both phases, but non-transoral applications, including combined transoral and transcervical, transcervical, and retroauricular access, appeared intermittently throughout the sequence rather than being restricted to a single early exploratory interval. **Figure 3** also demonstrates diversification of anatomic sites over time, with oral cavity cases becoming more frequent in the later portion of the series, consistent with the phase-based findings in **Table 3**.

### 3.6 Cumulative accrual of benign and malignant cases over time

**Figure 4:**
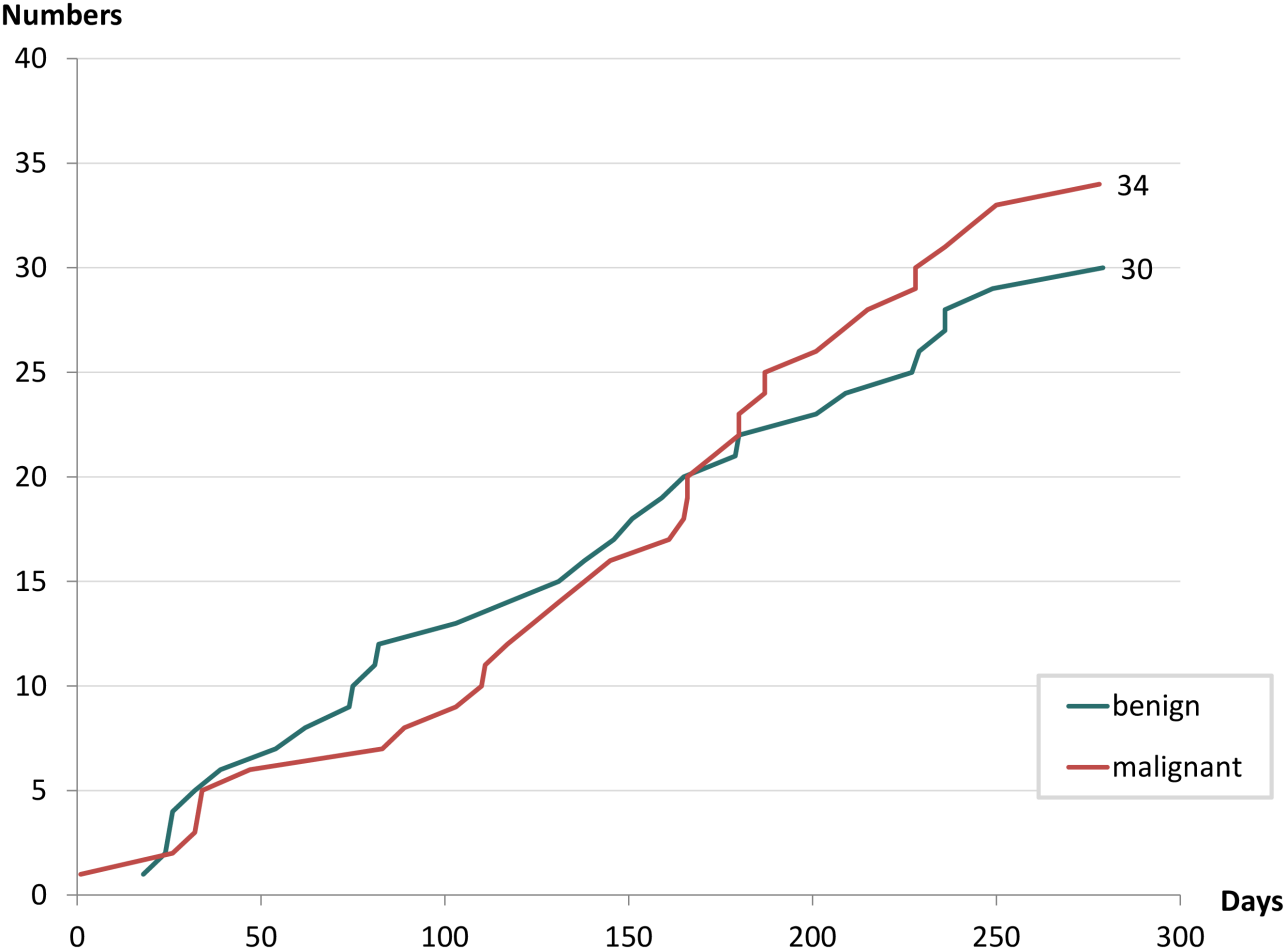
Cumulative accrual of benign and malignant cases over time. Cumulative case curves for benign and malignant lesions during the study period.

The temporal balance between benign and malignant case accrual is further illustrated (**Figure 4**). Throughout the implementation period, the cumulative curves for benign and malignant cases progressed in a broadly parallel manner, indicating that expansion of SP use occurred in both disease categories rather than being restricted to either benign or oncologic indications. Benign cases accumulated slightly earlier in the initial phase of the series, whereas malignant cases increased more prominently in the middle and later phases, ultimately reaching 34 cases by the end of the study period, compared with 30 benign cases.

## 4 Discussion

### 4.1 Early implementation and case accrual

The present study demonstrates the successful clinical integration of the da Vinci SP platform during a 9-month institutional transition, with 64 robotic head and neck procedures performed. Our early implementation findings highlight steady case accrual, supporting the SP system’s utility across a diverse range of anatomic sites and accommodating a balanced volume of malignant (53.1%) and benign (46.9%) pathologies. The observed parallel growth in both benign and malignant cases over time indicates that the new platform was broadly adopted rather than being restricted to a solitary oncologic or benign niche. This steady implementation reflects the established institutional learning curve associated with adopting transoral robotic systems, as emphasized in recent scoping reviews evaluating the learning process in transoral robotic surgery (5). Furthermore, it situates our transition within the broader shift toward single-port systems over traditional multi-port platforms in head and neck procedures (6).

### 4.2 Diversification of surgical access routes

A central highlight of our early implementation phase is the significant diversification of surgical access routes facilitated by the SP platform. While transoral surgery predictably remained the dominant approach (65.6%), the system was successfully adapted for combined transoral and transcervical (18.8%), transcervical alone (7.8%), and retroauricular (7.8%) access routes. This transition represents a notable shift from our historical multi-port robotic experience, which was almost exclusively restricted to standard transoral corridors for oral and/or oropharyngeal subsites (7–9). While conventional multi-arm platforms posed significant structural and external positioning challenges for extraoral or deep-neck access, the single-port configuration enabled our team to extend beyond traditional transoral boundaries into complex transcervical and retroauricular tissue spaces. The successful institutional adoption of non-transoral techniques, particularly the retroauricular approach, parallels emerging international experience with robotic systems for remote-access neck surgery and selective neck dissection (10–12).

### 4.3 Surgeon-specific adoption patterns

Our results also reveal distinct adoption patterns between the two primary surgeons, reflecting the SP system’s flexibility in accommodating varying case complexities and patient demographics. Surgeon 1 utilized the platform for an older patient population with higher rates of malignancy (64.7%) and exposure-related risk factors, employing broader access routes and performing all reconstructive procedures (35.3%). This aggressive application in complex, elderly populations aligns with recent evidence supporting transoral robotic surgery for head and neck cancer in older demographics (13, 14). Consequently, Surgeon 1 required a significantly higher rate of perioperative adjuncts, notably nasogastric tube placement (43.1%), a well-documented requirement following complex robotic head and neck resections (15). In contrast, Surgeon 2 adopted a more conservative initial approach, focusing exclusively on transoral access for primarily benign lesions (92.3%) in younger patients, requiring minimal adjuncts (7.7% nasogastric tube placement) and no reconstructive procedures. This reflects the system’s simultaneous suitability for less invasive, benign indications such as OSA (16).

These individualized utilization trends are best understood when contextualized within historical institutional practice profiles. Prior to the introduction of the SP system, both complex oncologic resections and benign operations, such as transoral tongue-base reductions for OSA, were already mature components of our multi-port robotic program. Consequently, Surgeon 1’s initial case mix did not reflect an intentional expansion of clinical boundaries, but rather a direct migration of their pre-existing high-volume workload onto the single-port platform. This initial cohort was characterized by advanced malignancies and older patients with significant exposure histories. In contrast, Surgeon 2’s initial focus on transoral access for younger patients with benign lesions represents a structured, stepwise phase-in strategy, allowing for ergonomic optimization in low-morbidity anatomical sites before broader clinical deployment. Framing these trends against baseline institutional multi-port data replaces subjective procedural adoption archetypes with an objective overview of individualized transition pathways that naturally reflect a surgeon’s established clinical volume and subspecialty focus.

### 4.4 Chronological evolution of the single-port platform

An analysis of the chronological evolution from the early (first 32 cases) to the later phase (subsequent 32 cases) further elucidates the platform’s maturity within the institution. While overall surgeon distribution and pathology behavior remained stable, case composition notably evolved over time. Specifically, oropharyngeal or tonsillar cases were more common in the early phase (53.1% vs. 34.4%), whereas oral cavity procedures became more prominent in the later phase, doubling from 25.0% to 50.0%. This expanding application to oral cavity tumors, which often present access challenges such as trismus, highlights the SP system’s feasibility and maneuverability in complex anterior corridors (7, 9). Concurrently, reconstructive procedures became more frequent in the later phase (34.4% vs. 21.9%), alongside numerical increases in combined plastic surgery with free flap reconstruction (18.8% vs. 6.3%).

Integration with pre-existing institutional practice volumes provides a clearer context for understanding this shifting case distribution. The marked increase in oral cavity cases and advanced reconstructive procedures during the later phase should not be viewed as an arbitrary escalation of procedural complexity. Instead, the trend mirrors a predictable return to the baseline case mix typically managed within our high-volume program before the technological transition began. Meticulous defect management following oncologic extirpation is highly dependent on advanced head and neck reconstruction, a domain where the growing integration of transoral robotic systems has been previously well documented (17).

As the surgical team optimized operating room setups and stabilized single-port docking mechanics during the early phase, they were able to safely transition standard multi-port protocols for complex anterior corridors and microvascular free flap reconstructions onto the new platform. A parallel progression of the cumulative accrual curves further confirms that single-port adoption experienced sustained, parallel growth across both benign and malignant indications rather than being restricted to a limited exploratory niche. Such temporal diversification is consistent with broader geographic and institutional patterns reflecting the evolving, multi-indication application of robotic-assisted head and neck surgery globally (18). Ultimately, initial single-port implementation is rapidly followed by a normalization of the pre-existing institutional case mix as initial technical constraints are resolved.

### 4.5 Ergonomic and workflow efficiencies

Beyond the expanding surgical indications and access routes, the rapid accrual of 64 cases over nine months underscores the ergonomic and workflow efficiencies inherent to the SP platform. As recently published data showed that during our initial institutional validation phase, the da Vinci SP system achieves efficient, consistent docking and draping times of 5 to 6 minutes, a logistical factor critical to maintaining high operative volume during a technological transition (4). The single-port design was developed to address the ergonomic and technical limitations traditionally associated with multi-port, single-incision surgery, thereby offering enhanced maneuverability in narrow anatomical spaces.

Recent literature heavily emphasizes that optimizing surgeon ergonomics and physical workflow is paramount for the sustainable, long-term adoption of robotic systems in head and neck practice (19). By reducing external instrument collisions and improving intraoperative console ergonomics, platforms that prioritize workflow efficiency enable a smoother surgical process than earlier multi-arm iterations (20). This enhanced ergonomic profile likely contributed to our institution’s ability to manage both straightforward transoral benign resections and complex, multi-route reconstructive procedures without overwhelming the surgical staff. Ultimately, these combined ergonomic and operational efficiencies supported the continuous case accrual, necessitating the installation of a second SP system at our facility (21, 22).

### 4.6 Functional outcomes and postoperative complications

Nevertheless, the transition to the SP platform warrants careful evaluation of functional recovery and safety profiles. Our data revealed that perioperative adjuncts, such as nasogastric tube placement (43.1% for Surgeon 1) and ICU stays, were utilized selectively and were primarily associated with complex reconstructive cases. Understanding these baseline complications and adjunct metrics, as presented in **Table 2**, is vital, as managing postoperative complications, such as hemorrhage (23), and minimizing acute postoperative pain (24) are central to optimizing patient recovery during institutional learning curves. Additionally, short- and long-term swallowing outcomes remain critical functional endpoints following advanced robotic resections and should be assessed in future prospective studies (25). The minimal adjunct requirements observed in our benign cases suggest that the enhanced maneuverability and precise tissue handling of the single-port system positively support these functional recovery pathways.

### 4.7 Single-port implementation in OSA

While Surgeon 2 used the platform almost exclusively for benign transoral cases, such as OSA, which comprised 92.3% of their sub-cohort, Surgeon 1 also contributed a baseline volume of complex sleep surgery to the overall series. Robust tracking of this benign caseload builds directly upon previously published institutional data evaluating single-port transoral robotic surgery specifically for head and neck lesions and airway obstruction (4). Implementation safety was underscored by the minimal need for perioperative support measures in these benign cases, with no instances of intensive care unit admission or tracheotomy. Sustained growth across both benign and malignant applications indicates that the single-port system provides a versatile solution across diverse head and neck practice models rather than being restricted to an early exploratory oncologic niche. Enhanced visualization and single-port deployment are particularly well-suited for safely navigating narrow anatomical corridors, enabling efficient instrumentation within the confined retrolingual space (26). Recent technical evaluations confirm the safety and reproducibility of single-port transoral tongue-base resections for airway expansion (27), supporting the role of standalone transoral robotic surgery in comprehensive upper airway management (16). The high volume of benign resections observed during this nine-month transition phase confirms that the platform can be safely and simultaneously integrated into routine sleep surgery programs alongside complex oncologic care.

### 4.8 Study limitations

The present study has several limitations. First, its retrospective, observational design at a single institution may limit the generalizability of the findings to centers with different robotic training paradigms, operative volumes, or resource allocations. As the indications for robotic surgery expand globally, adherence to standardized governance frameworks and guidelines for good practice is increasingly necessary to ensure reproducible outcomes across institutions (28). In addition, while our study tracked the specific use of perioperative adjuncts, future platform transitions would benefit from implementing more formalized, objective assessment tools for transoral robotic surgery to better quantify individual surgical performance and training progression during the learning curve (29). Second, while the cohort of 64 patients successfully demonstrates early implementation trends, ongoing case accrual, and access diversification over the 9-month period, it remains relatively small for extensive statistical subgroup analyses. The observed differences may alternatively be explained by unequal expertise and non-random case allocation. Finally, this study appropriately focuses on early perioperative outcomes and surgical access reconfiguration. Consequently, long-term oncologic efficacy, including verification of clear surgical margins compared with conventional open approaches, and functional outcomes in salvage surgery scenarios, were beyond the scope of this initial analysis (30, 31). Similarly, assessing extended functional recovery measures, such as comprehensive short- and long-term swallowing outcomes (25), and optimizing oncologic results in accordance with recent clinical guidelines (32), will require future longitudinal investigations as our institutional SP cohort matures.

## 5 Conclusion

The early clinical integration of the da Vinci SP platform at our institution demonstrates a highly successful nine-month transition from multi-port to single-port robotic head and neck surgery. Our cohort of 64 consecutive procedures highlights the system’s exceptional versatility in reconfiguring operative access pathways, allowing surgeons to adaptively expand beyond the traditional transoral route to include combined transoral and transcervical, transcervical-only, and retroauricular approaches. The platform successfully accommodated distinct surgeon adoption strategies, proving a safe and effective modality for both complex oncologic resections requiring major reconstruction and more conservative, standalone interventions for benign indications such as OSA. Moreover, the chronological evolution of our caseload, characterized by an expanding application to complex oral cavity tumors and an increase in reconstructive procedures over time, indicates a rapid institutional learning curve and sustained parallel growth across diverse pathologies. Ultimately, the da Vinci SP system offers a robust, highly maneuverable platform capable of safely meeting the comprehensive needs of a modern, high-volume head-and-neck surgical practice.

## Data Availability

The datasets presented in this study can be found in online repositories. The names of the repository/repositories and accession number(s) can be found below: Harvard Dataverse: Tsai S. Replication Data for Clinical Dataset of Robotic Head and Neck Procedures Performed During Early Single-Port Platform Adoption (2026), https://doi.org/10.7910/DVN/CLMJHR.

## References

[1] Mahfoz TB . Single-port versus multiport robotic surgery in head and neck procedures: a systematic review and meta-analysis of surgical parameters. Ann Surg Treat Res. (2025) 109:293–301. doi: 10.4174/astr.2025.109.5.293 41255476 PMC12621918

[2] Kim JK Kim DW Ryu JS Kang S Kim EJ Kang SW . Recent advances in single-port robotic thyroidectomy: evolution, techniques, and clinical outcomes. Ann Surg Treat Res. (2026) 110:3–11. doi: 10.4174/astr.2026.110.1.3 41541291 PMC12799353

[3] Tung MC Ou YC Tsai SCS Law KS Yu JK Huang LH . Multi-specialty application of the da Vinci SP surgical system: initial experience from a prospective observational study at a single hospital in Taiwan. Tungs Med J. (2025) 19:95–103. doi: 10.4103/etmj.etmj-d-25-00047

[4] Lin TS Pan CY Chen SW Nian MW Tung MC Tsai SCS . Single-port transoral robotic surgery for head and neck lesions and obstructive sleep apnea: a prospective real-world case series from Taiwan. Laryngoscope Investig Otolaryngol. (2026) 11:e70334. doi: 10.1002/lio2.70334 41623430 PMC12858423

[5] Lechien JR . Learning process of transoral robotic surgery for head and neck cancers: a scoping review. Oral Oncol. (2025) 171:107788. doi: 10.1016/j.oraloncology.2025.107788 41270524

[6] Choi JY . Single-port versus multiport robotic surgery in head and neck procedures: the emergence of a new platform. Ann Surg Treat Res. (2025) 109:283–5. doi: 10.4174/astr.2025.109.5.283 41255471 PMC12621785

[7] Pan CY Lin TS Lee TP Tsai SC . Transoral robotic surgery in oral tongue cancer patients with trismus: a retrospective evaluation of feasibility and surgical outcomes. Oral Oncol. (2025) 168:107597. doi: 10.1016/j.oraloncology.2025.107597 40818210

[8] Chang CC Chen CH Hsieh TL Chang KH Huang JY Lin FCF . Clinical characteristics and treatment outcomes of oral cancers using transoral robotic surgery in an endemic region. Cancers (Basel). (2023) 15:4896. doi: 10.3390/cancers15194896 37835589 PMC10571799

[9] Lin TS Luo CW Hsieh TL Lin FCF Tsai SCS . Transoral robotic surgery for oral cancer: evaluating surgical outcomes in the presence of trismus. Cancers (Basel). (2024) 16:1111. doi: 10.3390/cancers16061111 38539445 PMC10969054

[10] Bertelli AA Monazzi BV Jareño TT Barros Silva LA Guedes de Toledo Barros R Massarollo LCB . Robotic neck surgery using retroauricular approach - Experience of 60 procedures. J Robot Surg. (2025) 19:713. doi: 10.1007/s11701-025-02891-4 41139717

[11] Abdelwahab SI Taha MME Farasani A Moshi JM Assiri A Alshahrani S . Thematic evolution and global trends in retroauricular and robotic-assisted surgery: a comprehensive bibliometric review. J Robot Surg. (2025) 20:68. doi: 10.1007/s11701-025-03021-w 41364295

[12] Agarwal M Singh A Rai S Bansal K Singh P Karthik P . Retroauricular robotic neck dissection: step-by-step technique and feasibility in selected early OCSCC. Eur Arch Otorhinolaryngol. (2026). doi: 10.1007/s00405-026-10142-x 41862721

[13] Pamuk E Beharry A Lambercy K Dalla-Vale M Wahler N Hoşal Ş . Transoral robotic surgery (TORS) for head and neck cancer in the elderly population: functional outcomes, survival, and complications. Head Neck. (2026) 48:1016–30. doi: 10.1002/hed.70097 41257353 PMC12972643

[14] Russo E Porcuna DV Gorphe P Paleri V Pellini R Costantino A . Transoral robotic surgery for elderly patients with oropharyngeal and laryngeal cancer: a comprehensive review. J Clin Med. (2026) 15:1586. doi: 10.3390/jcm15041586 41753273 PMC12941629

[15] Jones O Silva P Winter S . Investigating the requirement for nasogastric tube feeding following transoral robotic surgery for head and neck cancer in Oxford: a retrospective cohort study. J Laryngol Otol. (2025) 139:496–500. doi: 10.1017/S0022215125000076 40136053 PMC12303716

[16] Chaidas K Mouratidou S . Standalone transoral robotic surgery for obstructive sleep apnoea: a systematic literature review of clinical outcomes. Life (Basel). (2026) 16:332. doi: 10.3390/life16020332 41752968 PMC12942123

[17] Song SS Lee ZH Yu JZ . Transoral robotic surgery (TORS) in head and neck reconstruction. J Clin Med. (2025) 14:5775. doi: 10.3390/jcm14165775 40869601 PMC12386521

[18] Gilmore D Michelle LR Hu X Kang SY Seim NB Haring CT . Geographic and institutional patterns of transoral robotic surgery in head and neck cancer. Head Neck. (2026) 48(6):1605. doi: 10.1002/hed.70179 41556601 PMC13155175

[19] Formeister E Pentico M Butts SC . Ergonomics. Otolaryngol Clin North Am. (2025) 58:965–82. doi: 10.1016/j.otc.2025.08.012 40976730

[20] Lingl JP Hoffmann TK Goldberg-Bockhorn E Greve J Boehm F . Exoscopic-assisted head and neck surgery: a comparative evaluation optimizing ergonomics and workflow. J Robot Surg. (2025) 19:391. doi: 10.1007/s11701-025-02551-7 40659860 PMC12259794

[21] Garavini AB de Carvalho Veludo AC Alexander K Cotte J Leslie S Thanigasalam R . Minimum caseload for cost-effective robotic-assisted surgery: a systematic review. J Robot Surg. (2026) 20:216. doi: 10.1007/s11701-025-03122-6 41634236 PMC12868110

[22] Keirns D Hsia B Guerra-Navarro PV Wu X Silberstein P Gardner JR . A comprehensive analysis of socio-economic and clinical factors in head and neck cancer patients receiving robotic surgery. J Laryngol Otol. (2025) 139:1062–9. doi: 10.1017/S0022215125102843 40702953

[23] Pinhorn C Stewart R Payne T Edwards D Singh-Dehal Y Muralitharan M . Haemorrhage following transoral robotic surgery in head and neck cancer. J Robot Surg. (2025) 20:133. doi: 10.1007/s11701-025-03018-5 41460401 PMC12748133

[24] Toppenberg AGL van den Bosch EW Plaat RE Halmos GB Wolff AP Schwandt LQ . Factors influencing acute postoperative pain after TORS. Acta Otolaryngol. (2025) 145:1175–80. doi: 10.1080/00016489.2025.2559881 41138230

[25] Weiland AC Samant S Clain AE Martin-Harris B . Short- and long-term swallowing outcomes in head and neck cancer patients receiving TORS and adjuvant therapy. Head Neck. (2025) 47:1345–54. doi: 10.1002/hed.28033 39714099 PMC12038223

[26] Phua CQ Lee G Lee LA Li HY Fang TJ Tsai MS . Navigating transoral robotic surgery for obstructive sleep apnoea. J Laryngol Otol. (2025) 139:1136–42. doi: 10.1017/S002221512510340X 40898711

[27] Fang TJ Lu YA Chuang LP Wanni-Lin Tsai MS . Evaluation of single-port TORS tongue base resection for obstructive sleep apnea: safety and patient outcomes. J Robot Surg. (2025) 19:520. doi: 10.1007/s11701-025-02699-2 40875140 PMC12394304

[28] Keenan RA O'Keeffe DA O'Neill A Fleming CA McVey R Moran T . Robotic surgery in Ireland: national governance framework and a guide to good practice. Surgeon. (2026) 24:31–8. doi: 10.1016/j.surge.2025.10.007 41203496

[29] Sezer D Demirel D Leake M Shafi MM De S Holsinger FC . Analysis and objective assessment of transoral robotic surgery. Int J Comput Assist Radiol Surg. (2026). doi: 10.1007/s11548-026-03605-3 41913026 PMC13183141

[30] Omura G Eguchi K Sakai T Ito T Tanaka A Katoh M . Feasibility of ensuring surgical margins of transoral robotic surgery for lateral oropharyngectomy for p16-positive oropharyngeal squamous cell carcinoma patients compared with non-robotic surgery: a single-center, retrospective study. Auris Nasus Larynx. (2026) 53:107–10. doi: 10.1016/j.anl.2025.12.016 41512815

[31] de Groot ECM Nyirjesy SC Faden DL Lin DT Deschler DG Feng AL . Salvage transoral robotic surgery with submental flap reconstruction: functional and oncologic outcomes. Ann Otol Rhinol Laryngol. (2025) 134:797–805. doi: 10.1177/00034894251347103 40539857

[32] Safavi AH Riaz N Sherman EJ Wong RJ Lee NY . Optimizing outcomes after transoral robotic surgery for oropharyngeal carcinoma: reflections on the ASCO guideline. J Clin Oncol. (2025) 43:2655–6. doi: 10.1200/JCO-25-00654 40532133

